# Nurturing environments for shaping trauma-responsive care and recovery: a realist coproduction study protocol

**DOI:** 10.3389/frcha.2026.1816036

**Published:** 2026-10-08

**Authors:** Sarah Parry, Geoff Wong, Steven Ariss, Sadie Rodell, Elaine Craig, Emma Ford, David Graham, Zarah Eve, Joy Duxbury

**Affiliations:** 1 Faculty of Biology, Medicine and Health, University of Manchester, Manchester, United Kingdom; 2 Pennine Care NHS Foundation Trust, Manchester, United Kingdom; 3 Nuffield Department of Primary Care Health Sciences, University of Oxford, Oxford, United Kingdom; 4 Faculty of Health & Education, Manchester Metropolitan University, Manchester, United Kingdom; 5 Stockport Metropolitan Borough Council, Greater Manchester, United Kingdom; 6 The Care Leavers' Association, Manchester, United Kingdom; 7 Manchester Metropolitan University, Manchester, United Kingdom; 8 University of Cumbria, Ambleside Campus, Cumbria, United Kingdom

**Keywords:** children's home, coproduction, realist evaluation, residential children's homes, trauma-responsive care

## Abstract

**Background/rationale:**

Children and young people in residential care often face multiple, complex traumas, including the distress of being removed from familiar environments. Trauma-responsive care (TRC) offers a framework for understanding and responding to these experiences, yet there is no standardised TRC intervention specifically designed for children's homes in the UK. This lack of tailored guidance contributes to inconsistent care quality and limited support for staff and service providers.

**Objectives/aims:**

The NEST Study aims to co-produce, pilot, and evaluate a trauma-responsive care intervention for children's homes through co-production and realist methods. NEST seeks to understand how, when, and why TRC interventions work for cared-for children and to develop an implementation toolkit that supports sector-wide improvement.

**Study design overview:**

This multi-phase realist co-production study comprises three workstreams: 1. A Rapid Realist Review to develop an initial programme theory of TRC in residential settings. 2. Experience-Based Co-Design (EBCD) with children, staff, and stakeholders to co-create the intervention, in alignment with the programme theory. 3. A Realist Evaluation of the intervention across six children's homes in Northern England, followed by refinement of the implementation toolkit.

**Methods:**

Up to 150 children and young people, 60 staff, and 30 stakeholders will participate. Mixed methods will include qualitative interviews, creative participatory techniques, and validated and adapted quantitative measures (e.g., SDQ, RCADS, ProQOL). Quantitative data will be analysed using descriptive statistics and regression modelling. All data will be analysed using realist logic to identify, refine and test context-mechanism-outcome configurations. The intervention will be piloted over 12 months. The interpretation of emerging findings will be supported by a Lived Experience Advisory Group and Stakeholder Advisory Board.

**Key outcomes and analyses:**

Anticipated outcomes include a refined realist programme theory of TRC in homes for cared for children and an implementation toolkit for care providers to use. Wellbeing and quality of life measures will provide insights into the impact of the pilot for the children, young people and staff who experience the trauma-responsive care pilot throughout the evaluation process.

**Clinical Trial Registration:**

identifier ISRCTN17921858.

## 1 Introduction

### 1.1 Background

Children in care in children's homes are extremely vulnerable and the care system, according to the latest independent government enquiry, is “fragmented” and “dysfunctional” (1), leading to poor outcomes for care experienced young people. Children in care are five-times more likely to be excluded from school compared to their peers (2, 3). Looked after children are four-times more likely to experience mental health difficulties (4, 5). Education breakdown and involvement with the criminal justice system is common (6–9) with 39% of children in secure/youth forensic centres being looked-after (10).

Care experienced young people have experienced multi-type traumas and evidence indicates they need consistent, high-quality trauma-responsive care (TRC) (11);. However, no tailored intervention for the unique setting of children's homes exists. There are currently 82,000 children in care in England (12), 63% of whom enter care due to abuse and neglect. These children then experience inconsistent care. Part of the fragmentation across the sector is due to rapid privatisation. 80% of children's homes are now run by independent companies. Local authorities run 14% of homes offering 16% of placements, and voluntary organisations offer 4% of homes (13). Private providers charge an average of £3,830 a week per child and the quality of care they provide is variable and sometimes opaque. Local authorities continue to face financial challenges and the demand for suitable placements outstrips supply.

12,898 young people are housed in children's homes, cared for by paid staff who work on a shift basis. Residential care aims to promote recovery, rehabilitation, and treat social, emotional, and behavioural difficulties. However, children's homes can be hostile environments for vulnerable young people and under-supported staff; sometimes referred to as “placements of last resort” (14).

TRC is highly relational and therapeutic. The independent review (1) states a desire for residential childcare to incorporate TRC into care provision to improve outcomes and create a shift towards needs-focused relationship-based care. Therefore, our project is both aligned to and timely within the national conversation and priorities for reform for children's homes.

Care providers in children's homes need to be guided by the principles of TRC and have a tailored transparent TRC implementation toolkit for the wellbeing and safety of children and frontline staff (14). The five critical factors for the successful implementation of TRC in residential settings are (15):

1. Senior leadership commitment,
2. Sufficient staff support,
3. Amplifying the voices of children and families,
4. Aligning policy and programming with trauma responsive principles,
5. Using data to help motivate change.

Our project has already amplified the voices of young people and stakeholders through extensive PPIE, informing the design of this study. Their voices will continue to be central. Our dissemination plans speak directly to informing policy and using our data to motivate change. Our primary research has laid solid foundations for developing staff support (16, 17) and we have worked closely with service leaders to ensure buy-in.

Building further on primary and secondary research we have undertaken (17–19), alongside extensive PPIE, we will co-design, pilot, evaluate and refine the first much needed children's home specific trauma-responsive care intervention, implementation and evaluation toolkit with the aim of transforming care transparency and quality. Our approach for this complex intervention has been informed by the latest MRC Guidance (20).

The limited research available into the psychosocial impact of being looked-after has established there are significant concerns about the safety and effectiveness of residential care (21, 22). Research consistently demonstrates residential care is associated with low educational attainment, high unemployment rates, poor physical and mental health, early/unplanned pregnancy and parenthood, homelessness, justice involvement, and social isolation (14, 19, 23). Looked-after children are amongst the most vulnerable in society (21, 22, 24), in childhood and adulthood (25–27). There is a clear need for reform and effective intervention to reduce the impact of childhood adversities and likelihood of future re-victimisation (28).

The implementation of TRC requires research attention, so that TRC can be delivered in an optimal and tailored manner in children's homes. The value added of this project is that the new TRC intervention with an implementation toolkit will be co-produced with young people and staff, then evaluated in real-life settings, demonstrating how, when, for whom and to what extent TRC can be implemented across children's homes.

Research Design Service funded PPIE consultations with care providers, frontline workers, care experienced young people and the Policy Team of the Department for Education between 2020 and 2022 identified a significant gap in knowledge and evidence for TRC in children's homes.

### 1.2 Problem statement

While TRC is increasingly recognised as essential for promoting emotional safety, preventing traumatisation, and improving the stability of placements, there is currently no standardised intervention tailored specifically for homes for cared for children in the UK. This absence of structured guidance leaves care providers without a clear framework for implementation, resulting in variable care quality and limited support for staff. Moreover, existing TRC models are often designed for clinical, judicial or educational settings and do not adequately address the unique relational, organisational, and developmental needs of children in residential care. There is a critical need for a co-produced, contextually responsive TRC intervention that reflects the lived experiences of young people and frontline staff, and that can be feasibly implemented across diverse care settings. The nurturing environments for shaping trauma-responsive care and recovery (NEST) Study addresses this gap by developing and evaluating a trauma-responsive intervention and implementation toolkit specifically designed for children's homes, informed by realist methodology and participatory design.

### 1.3 Rationale for the present study

There is an urgency to ensure safe and theoretically informed TRC is available, with a greater degree of parity and transparency across services. The new TRC intervention and implementation toolkit will provide clear guidance on governance, staff training, child centred and needs led care, supervision, therapeutic approaches to care, continual improvement, measuring developmental outcomes and therapeutic quality.

#### 1.3.1 Adversity and trauma-responsive care in children's homes

A significant body of evidence has consistently indicated increased exposure to adversity affects neurological, physiological, emotional, and immunological health and development (25, 29–31). Although early adversities can have a lasting impact for children and young people, children living in out-of-home care experience better outcomes when care providers can be emotionally available. Perhaps for this reason, evidence suggests TRC has a positive impact for young people, as it positively reinforces the importance of caring for frontline staff, which can increase their capacity for emotional attunement.

Frontline practitioners in children's homes hold positive attitudes towards TRC and support its implementation (32). There is potential for TRC approaches to protect against stress and trauma experienced by care staff. In turn, over time, this could increase job satisfaction, reduce high staff turnover, improve wellbeing and enhance ability to provide stable, caring relationships. Across the workforce, there is an understanding of the implicit connection between the principles of TRC and the past experiences of the children in their care. TRC principles align with high value behaviours across the profession, such as empathy-focused communication (33). Further, children's wellbeing and placement stability improve with increased exposure to TRC over time. Importantly, no one staff member or caregiver is central to implementing TRC, highlighting the need for organisational buy-in. Finally, non-clinical staff can effectively implement TRC (34), meaning TRC is both an effective and feasible approach to improve stability, wellbeing and outcomes for social care staff in children's homes.

#### 1.3.2 Trauma-responsive care and mental health

TRC is an approach that recognises the impact of adversity upon a person, rather than locating difficulties within the person. For example, it is well documented that symptoms of chronic stress, common for children in care, can include hypervigilance, difficulties in attention, fatigue, sleep difficulties and hyperactive fight-or-flight responses; symptoms also associated with ADHD. Children in care are three-times more likely to be diagnosed with ADHD than non-looked after children (35). If a child's presentation is perceived through a lens of ADHD, “naughtiness” or simply “untreatable”, the child's care will reflect this. Other common diagnoses for children in care include Oppositional Defiant Disorder, Conduct Disorder, Anxiety Disorders and Unspecified Functional Psychosis. These diagnostic terms do not communicate a child's mental health needs effectively in social care environments or explain how a traumatised child might communicate their distress and needs (e.g., through violence, self-harm, or withdrawal). These terms do not inform how social care practitioners can support mental health recovery in residential homes. However, if a child's difficulties are understood as a consequence of trauma, the focus can more easily be placed upon compassionate therapeutic care to aid recovery.

TRC also requires the organisation, not only individual practitioners, to work with trauma-sensitivity and emotional availability, with suitable support in place for frontline carers. TRC can improve engagement, adherence, and health outcomes, as well as staff wellness (36).

The complex needs of young people in children's homes have not contributed to the development of existing TRC interventions, which often lack the systemic elements required for operationalisation in children's homes (37). A tailored and holistic intervention for child-focused TRC that recognises adversity, resilience and children's homes dynamics is needed. SP has recently undertaken a systematic review of mechanisms that support resilience and post-traumatic growth for young people in residential care, drawing upon 27 international studies (18), which has further highlighted the need for a new TRC intervention.

Organisations providing TRC need to create a safe space, empower service user involvement, identify trauma-related needs at individual and systemic levels, nurture a culture of wellbeing and resilience for individuals and the organisation as a whole, working with a “whole systems” approach, supporting cross-agency collaboration and integrating communication (38–40). Providers can face barriers in terms of delivering TRC due to difficulties in care coordination between an extensive range of health, social care and educational organisations (41). Often, competing demands, complex needs, and a lack of national guidance for these providers outweigh available resources, which can result in suboptimum standards of care.

##### 1.3.2.1 Aim

To co-produce, pilot, and evaluate a new trauma-responsive care intervention for use in children's homes to promote wellbeing and recovery.

##### 1.3.2.2 Research question

How, when, and why can a theoretically informed evidence-based trauma-responsive care intervention, tailored for children and young people in children's homes, improve care quality and experience?

##### 1.3.2.3 Objectives

1. Co-produce a new tailored intervention for children's home care through a rapid realist review (Ws1) and experience-based co-design (Ws2).
2. Pilot and evaluate the intervention across local authority and privately run homes in Greater Manchester, using a realist evaluation approach to identify how, why, and in what contexts the intervention could support the recovery and wellbeing of young people in care (Ws3a).
3. Develop an implementation toolkit for wider use and further evaluation (Ws3b).
4. Promote positive change for children's homes and vulnerable young people through an appropriate dissemination, impact, and communications strategy.

## 2 Materials and methods

### 2.1 Study design

The NEST Study is a multi-phase realist co-production development study comprising three workstreams:

1. Workstream 1: A Rapid Realist Review to develop an initial programme theory of trauma-responsive care (TRC) in residential settings.
2. Workstream 2: Experience-Based Co-Design (EBCD) with children, staff, and stakeholders to co-create the intervention.
3. Workstream 3: Realist Evaluation of the intervention across six children's homes, followed by refinement of an implementation toolkit.

This is a mixed-methods, non-randomised, non-CTIMP study incorporating qualitative, observational, and pilot evaluation components. It is designed to develop and evaluate a complex intervention using realist approaches.

#### 2.1.1 Setting

The study will be conducted in six children's homes in Greater Manchester, UK: four local authority homes and two privately run homes in the Northwest of England (Figure 1).

**Figure 1:**
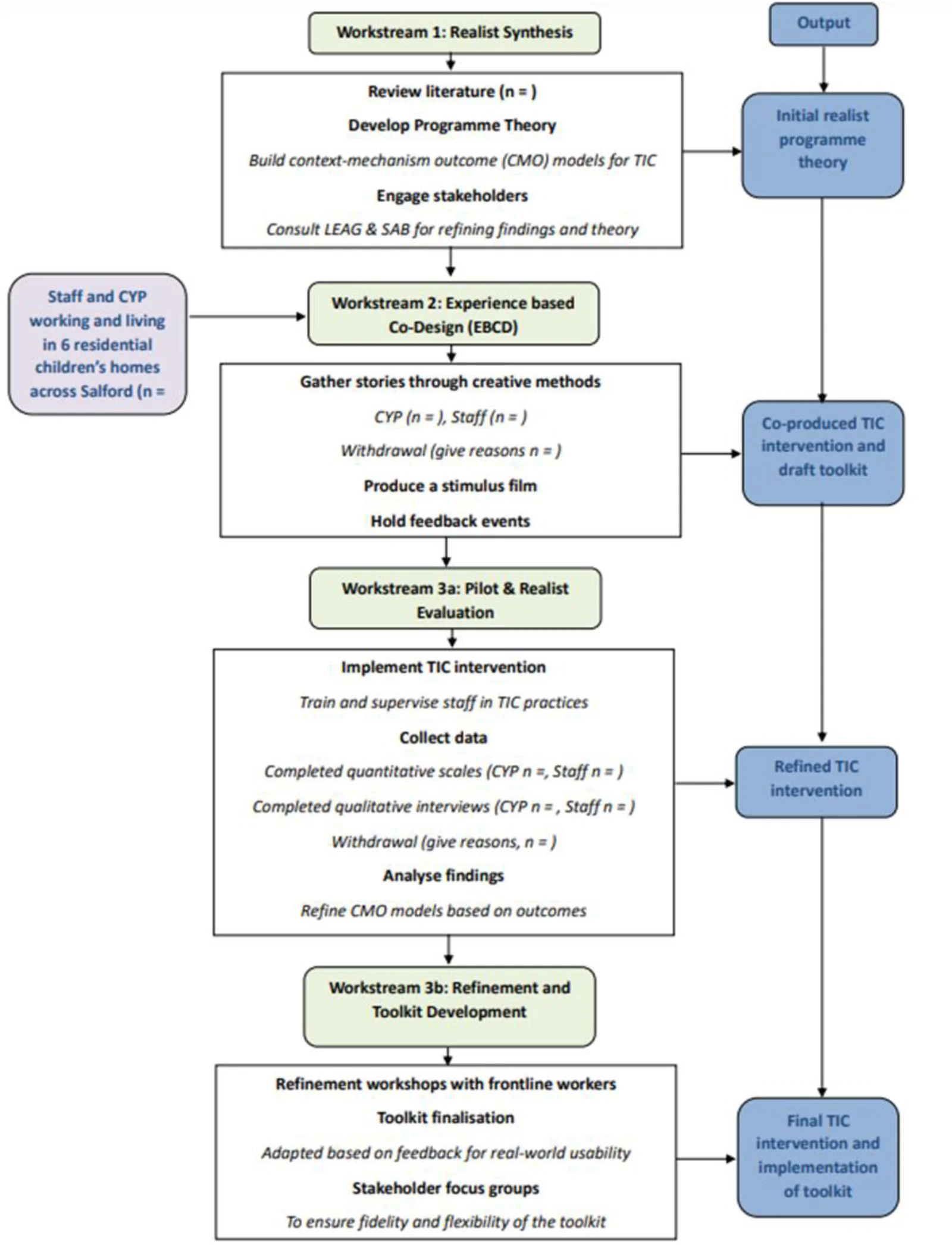
Recruitment through the phases of the nurturing environments for shaping trauma-responsive care and recovery (NEST) study.

#### 2.1.2 Eligibility criteria

Inclusion Criteria:

- Children and young people aged 11–18 residing in participating homes.
- Residential care staff, home managers, social workers, and stakeholders involved in care delivery.

Exclusion Criteria:

- Individuals not connected to the six homes.
- Children under 11 years of age.
- Young people under 16 will not be involved as stakeholder advisors.

### 2.2 Recruitment strategy

Following consent, the researchers will conduct semi-structured interviews according to the draft topic guides included with this NHS ethics/health research authority (HRA) application. The topic guides used to inform the qualitative interviews will be a living document, updated according to emerging findings from earlier interviews, new published literature in this area and feedback from our ongoing stakeholder consultations.

Interviews may take up to 90 min, depending upon how much or little participants wish to say, and will be recorded using encrypted recording devices and software. Participants will be asked if they would like to receive copies of their interview transcripts and a summary of the emerging findings of the study, for the purposes of ensuring accuracy and contribute to “member checking” procedures to ensure the trustworthiness of the study findings.

#### 2.2.1 Keeping in touch calls

To promote retention in the study, the research workers will contact the link person at each of the homes approximately two weeks before each data collection point and then again, a few days prior. These brief telephone calls will be an opportunity for the research workers to remind participants (usually staff) of upcoming research engagement and to resolve pragmatic barriers that may delay or hinder the participant's timely engagement in the follow-ups.

The study aims to recruit:

- Up to 150 children and young people
- Up to 60 staff
- Up to 30 stakeholders No formal power calculation is required due to the developmental nature of the study. Quantitative analyses will use descriptive and exploratory methods appropriate for pilot data.

#### 2.2.2 Consent procedures

##### 2.2.2.1 Assent and consent procedures

Where young people are in the care of the local authority, depending upon the nature of the care order, consent for a young person's participation will be gained from the placement provider, social worker, or child's named parent/caregiver, as appropriate. Following enrolment and the provision of informed assent or consent, participants will be invited to complete a questionnaire capturing relevant demographic information (e.g., age, gender, ethnicity, family circumstances) and a brief clinical history, including previous service use and any comorbid diagnoses.

##### 2.2.2.2 Participant engagement process

The research study will be introduced to potential participants either in person, via Microsoft Teams, or by telephone, depending on individual preferences and the appropriateness of the setting. Typically, engagement with young people will occur face-to-face, while meetings with staff are more likely to be conducted remotely for convenience.

Consent will be obtained from participants aged 16 and over, while assent will be sought from those aged 15 and under, alongside parental or caregiver consent. Initial consent/assent discussions will take place within each residential home. As new staff or young people enter the home environment, assent/consent will be sought at an appropriate time, in consultation with home managers.

Upon enrolment, participants will complete a demographic questionnaire (Laurens et al., 2020). Additionally, pen portraits will be developed for each participating young person using records held by the placement provider.

Baseline (T1) data will be collected following consent/assent. At each assessment point, young people will be asked whether they prefer to speak with the researcher alone or in the presence of a trusted adult (e.g., caregiver, social worker, mentor, advocate, key worker). Staff will similarly be given the option to speak privately or with their child present. This process will be consistently applied at all data collection stages (see Table 1). The taking of assent and consent will be managed by members of the research team. Where staff in the homes gather assent and consent from children and young people, members of the research team will support them throughout the process.

**Table 1:** Administration of measures.

Measure	Frequency	Time Point (Months)
Children and Young People		
Demographics	Once	1
Routine Monitoring Data (RMD)	Monthly	1–12/end of placement
RCADS	Twice	1, 12/end of placement
Bounce Forwards Scale	Twice	1, 12/end of placement
KidScreen	Three times	1, 6 & 12/end of placement
Emotional Support Scale	Twice	1 & 12/end of placement
Care Environment Scale	Twice	1 & 12/end of placement
Interviews/Reflective Group Discussions	Twice	1 & 12/end of placement
Residential Staff		
Demographics	Once	1
ProQOL (Self-assessment)	Twice	1, 12/end of employment
TRC Implementation Domains Tool, Qualitative interviews	Twice	1, 12/end of employment
Implementation Monitoring Template, Narrative Data	Bi-monthly	2, 4, 6, 8, 10, 12 during RPGs
Qualtrics Written Feedback, anonymous	Ongoing	Optional feedback loop throughout pilot
Social Workers		
Implementation Monitoring Template, Narrative Data	Bi-monthly	2, 4, 6, 8, 10, 12 during RPGs
Qualtrics Written Feedback, anonymous	Ongoing	Optional feedback loop throughout pilot

##### 2.2.2.3 Additional consent provisions

The study's consent form will include optional items relating to the collection and use of participant data. It will be clearly stated that these options are voluntary and that declining them will not affect participation in the study.

Given prior experience and anticipated media interest, participants aged 16 and over will be invited to separately consider consenting to receive information about potential media opportunities (e.g., radio or television interviews). This consent will be distinct from research participation consent, ensuring clarity and autonomy. Previous studies have shown that participants often value the opportunity to share their experiences publicly, hence this option is included. Participants who express an interest in hearing about media opportunities will be provided with clear information about the nature of the activity, including any potential implications for privacy and public identification. They will be encouraged to discuss any questions or concerns with a member of their care team before speaking with any producers from the media company. Participation in media activities will be entirely voluntary and separate to the NEST study procedures, overseen by the care provider and young person's legal guardian(s), and participants may withdraw their interest at any stage prior to engagement without affecting their involvement in the research study.

All participants will also be asked to consider providing consent for the following:

1. Audio recording of some assessments for quality assurance and procedural improvement.
2. Use of recordings for supervision, teaching, and training purposes.
3. Use of anonymised data for secondary research analysis.
4. Sharing anonymised data with other research teams.
5. Future contact regarding related research studies.
6. Future contact to receive a summary of study findings.
7. Documentation of study participation in clinical records.

##### 2.2.2.4 Withdrawal of consent and criteria for withdrawal

Participants may withdraw from the study at any time. If withdrawal occurs prior to the creation of the Experience-Based Co-Design (EBCD) collective child's voice film, all data will be deleted and excluded from analysis and dissemination.

If withdrawal occurs after the creation of the EBCD films (both age-specific and combined), the participant's data will be fully anonymised. This may involve removing the participant from the films entirely, concealing or blurring their identity, and replacing their voice with an actor's voiceover to ensure anonymity.

Participants who lose the capacity to provide informed assent or consent during the study will be withdrawn. Withdrawal may also occur if a participant poses a significant safety risk to the research team or engages in aggressive or abusive behaviour. Such decisions will be made in consultation with clinical colleagues on a case-by-case basis.

Informed assent and/or consent will be obtained prior to qualitative interviews. Participants will be offered multiple options for confirming and documenting their consent, depending on the mode of contact:

1. Signing a hard copy of the consent form during face-to-face meetings.
2. Returning a signed hard copy via pre-paid post.
3. Returning a signed electronic copy via email.
4. Providing audio-recorded consent, captured using an encrypted device and stored separately from research data.

Either written or audio-recorded consent will be obtained. For minors, assent and parental consent will be required. Consent will be treated as an ongoing process throughout the study.

All research activities involving data collection will commence only after receiving NHS Ethics and Health Research Authority (HRA) approval, as well as local Capacity and Capability approval from participating NHS Trusts. The study will be conducted in full compliance with relevant legal requirements, the Declaration of Helsinki, Good Clinical Practice (GCP), and the UK Policy Framework for Health and Social Care Research (2017).

Where young people are under the care of the local authority, consent for participation will be obtained from the appropriate party, placement provider, social worker, or named parent/caregiver, depending on the nature of the care order.

##### 2.2.2.5 Description of intervention

The intervention will be a co-produced trauma-responsive care (TRC) framework tailored for children's homes. The tailored TRC protocol will be developed further over the process of the realist review and EBCD work, but is likely to include:

- Staff training in trauma-responsive theory and practice
- Trauma-responsive supervision and team formulation
- Organisational governance structures rooted in TRC principles and practices
- A practical implementation toolkit for day-to-day use in practice by all employees of the homes.

### 2.3 Procedures and materials

#### 2.3.1 Realist review

A rapid realist review will identify how, why, for whom, and in what contexts TRC that includes consideration of adversity and resilience *works* for children in residential care. TRC is a complex intervention, and an initial scoping review has revealed the literature is heterogenous, hence a realist review is appropriate. The realist review protocol will be registered with PROSPERO.

Our review will follow RAMESES quality and reporting standards (42). We will synthesise evidence from diverse sources (i.e., academic/grey literature, policy, related theory) to build an initial programme theory of a recovery-focused TRC intervention for children's homes. We will be able to achieve this within the timeframe as we have already conducted primary and secondary research into TRC, resilience, and post-traumatic growth. The review will therefore be informed by publications such as the “Using Logic Models Grounded in Theory of Change to Support Trauma-Responsive Initiatives” (43), which is focused upon children and families. Our job will not be to reinvent TRC, but to extrapolate understanding from relevant literature in relation to TRC and children's homes to formulate an initial programme theory.

Our five-step rapid realist review is adapted from Pawson's suggested steps, achievable in our given timeframe because our focus is very narrow; to develop an initial, rather than a highly detailed “finalised”, programme theory, building upon the aforementioned developments in this field.

Step1: Locate existing theories and define the review scope—Translate existing literature into concepts that will inform our initial programme theory by holding workshops with all members of the project team, SAB and LEAG, using their content expertise to generate potential realist causal explanations, which fit with the existing theory of change in relation to TRC, but specific to children's homes.

Step 2: Search for evidence—Undertake highly focused searches of electronic databases (e.g., CINAHL, PsycINFO, Medline, Embase, AMED) and keyword searches on Google Scholar, NIHR and other relevant websites to identify documents that contain data to refine the initial programme theory developed in Step 1. We will identify relevant policy/practice guidance from online searches of NHS, social care, and mental health service websites. We will download documents to bibliographic software.

We will include documents that: i) focus on TRC for young people; ii) consider good quality care in children's homes; iii) are available in English language. During the screening process, we will identify and flag documents that will be useful for other aspects of the programme, such as implementation or evaluation-focused literature, or influential documents about TRC more generally. These will also be used to identify data to address specific gaps or refine specific aspects of the initial programme theory.

Step 3: Select articles—To ensure consistent screening, all documents will be screened by SA and approximately one-third of all documents will be double screened by other members of the review team. The process will be coordinated by a senior researcher (SA) and inclusion/exclusion criteria will be iteratively developed and refined through regular communication in response to any disagreements or ambiguities. All documents not eliminated using information available in the title and abstract will be screened using the full text.

The review team will select and appraise documents included at the full text stage, applying the following criteria (44), in addition to the original inclusion/exclusion criteria:

- Relevance: Are sections of text within this document relevant to programme theory development?
- Richness: Can the document meaningfully contribute to theory development or testing?
- Rigour: Are these data sufficiently trustworthy to contribute to programme theory development?

Relevance will be appraised according to inclusion of three elements of TRC; Workforce Development, Trauma Focused Services, and Organisational Environment & Practices (45). Documents scoring “low” for relevance will be excluded at this stage. All remaining documents will be used for data extraction.

Step 4: Extract data—The review team will extract data from relevant documents as follows: i) descriptive data describing the included documents (e.g., date, type of document, study design, key findings etc.), which will be tabulated in Excel; ii) a separate Excel tab will be used to extract and construct theories, using one line for each discrete theory. Initial interpretation of data will be carried out at the extraction stage. Extracts of relevant data from full text documents, will be converted into causal theory statements, using “if, then, leading to, because” structure (where appropriate) and named for quick reference. At this stage (and subsequently as synthesis progresses), any substantive theories that support the programme theory (e.g., attachment theory) will be noted, and the level of confidence in the theory (high, medium or low) will be recorded along with a rationale for this judgement. To check for systematic errors, a minimum of 30% of documents will be extracted by 2 members of the research team and refinements or additions agreed through regular communication. Previously extracted data will be reviewed considering pertinent refinements to the extraction process.

Step 5: Synthesise data—SA will lead data analysis and synthesis with regular discussions with the research team to challenge and debate interpretations. The extracted causal theories, in the form of “if, then, leading to, because” statements will be categorised into themes (e.g., organisational culture, staff wellbeing etc.) and subthemes. A realist logic of analysis will be applied to these themes to develop potential context-mechanism-outcome configurations (CMOCs). We will use the following forms of reasoning to make sense of the data:

- Juxtaposing data: for example, where data about behaviour change in one source enables insights into data about outcomes in another source.
- Reconciling data: where data differs between apparently similar circumstances, it is appropriate to seek potential explanations for these differences having occurred.
- Adjudicating data: in terms of methodological strengths or weaknesses.
- Consolidating data: where outcomes differ in particular contexts, constructing an explanation of how and why these outcomes occur differently.

The resulting CMOCs will explain when, why, how and for whom the effects and experiences of trauma are influenced by TRC approaches, leading to intended (and unintended) outcomes in children's homes. To operationalise this logic, we will ask these questions:

1. Interpretation of meaning: do the contents provide data that may be interpreted as functioning as context, mechanism, or outcome?
2. Interpretations and judgements about context-mechanism-outcome configurations (CMOCs) e.g., what is the CMOC for the data that has been interpreted as functioning as context, mechanism, or outcome?
3. Interpretations and judgements about programme theory e.g., how does this CMOC relate to the initial programme theory?

##### 2.3.1.1 Engaging the LEAG and SAB

In facilitated online meetings, we will share, discuss and prioritise the developing findings (based on the CMOCs) with the LEAG and SAB groups. These consultation events will present findings appropriate to the experience and expertise of the members, will be open to all members from both advisory groups. Notes from discussions will be collated and discussed by the research team to identify if there are key areas of divergence and/or agreement with the developing programme theory. This deliberation process will allow us to assess how far and where findings may resonate with group members' diverse experiences and assumptions. Young people and non-professional carers will be remunerated for their time according to INVOLVE rates. Emergent findings will be presented to wider stakeholder groups at dissemination events and feedback will be sought. These engagement and consultation events will also be used to identify gaps in the evidence and inform the prioritisation of theory development regarding the long-term aim of developing and testing TRC interventions in children's homes.

**WS1 Output:** a realist initial programme theory of the intersections between the effects and experiences of trauma, TRC, adversity, resilience and residential care, which will iteratively feed into the development of the new intervention in WS2, informing likely key measures and topics for further investigation.

#### 2.3.2 Experience based co-design

Experience Based Co-Design (EBCD) is a participatory action research approach that draws upon creative and inclusive design tools and ways of thinking to bring practitioners and experts-by-experience together to improve quality of care (46). The initial programme theories and prioritised themes from WS1 will form an enquiry and development framework to guide the co-design process. EBCD enables the development of a touchpoint/stimulus film that amplifies the child's voice, enables professionals to view the service through young people's eyes, and stimulates discussion to identify priorities for change and intervention. To achieve this, we will conduct the following 8 stages:

Stage one: Interview 10 young people aged 16–18 from the homes to source stories of their experience. Locations and privacy will be arranged on an individual basis. PPIE suggests mealtimes and private spaces within the homes will be preferred for this age group.

Stage two: Co-produce 10x individual case study films with the participants aged 16–18 that will firstly be edited into individual case study films; secondly, into an age specific EBCD collective voice film; finally, it will be integrated into the EBCD collective voice film with all age range data. To do this, participants will have four choices over the level of anonymity they wish to have in the interviews. They can choose: 1) a face to camera filmed interview (participants face and voice are captured), 2) concealed face interview (the participants face will be disguised but the voice will be their own), 3) concealed face and actors voice interview, 4) audio interview only (no filming). If participants change their mind and would like to be anonymised at a later date, this choice can easily be amended during the editing prior to the film being shown. All participants will be given the opportunity to co-create their individual films with the film editor (EC). This is to ensure they are happy with how their experience is portrayed and give them the opportunity to edit out anything they do not want to be shared.

Stage 3: Employ creative methods and research-informed conversations with 10–15 children aged 11–15 in the homes, e.g., photographs, poetry, Lego, modelling clay, and artwork to elicit their experiences. This process will be accompanied by a graphic illustrator to record and theme discussions and outputs into a cohesive visual story, accessible for the children. PPIE suggests mealtimes and facilitated play times within the homes will be preferred for this age group, although 1-2-1 opportunities will also be available. We will then collate, anonymise then edit the young people's data (inclusive of pictures of visual outputs) into an age-specific EBCD collective voice film for participants aged 11–15 years.

Stage 4: Co-produce the final EBCD film by collating all the filmed data of both age ranges to be integrated into the final EBCD collective child's voice film.

Stage 5: Show the films to young people and frontline workers from the homes (*N* = 40). During this we will gather responses from the children through message cards, research-focused conversations over mealtimes, and provide the option of 1-2-1 research conversations with a member of the research team. We will also conduct 1-2-1 interviews with staff and older children where they prefer, to gather a rich understanding of their response to the stimulus film and broader perspectives and analyse all data to formulate an overarching narrative.

Stage 6: Present the overarching narrative and the findings of WS1 and WS2 through EBCD feedback events in each of the four homes, inviting reflective feedback, which will be audio recorded.

Stage 7: Collate feedback from the four events into the final emancipatory narrative, which will inform the key components for the intervention.

Stage 8: Co-ordinate intervention development workshops in the four homes to finalise and name the intervention and draft the initial toolkit, which will be further developed from experience of the pilot in WS3.

Participant remuneration: Based on conversations with colleagues in the local authority, remuneration will be agreed with service providers and house managers as appropriate at each stage of workstreams two, three and four. Where suitable and appropriate, INVOLVE rates will be followed and vouchers will be offered. However, there may be other occasions where it is more appropriate for our PPIE budget to support a group outing or activity (e.g., cinema or bowling trip) for the young people in a home who have taken part in a research activity in recognition of their time and contribution. Funding will be made available for catering and craft activities to support the research activities as needed. This flexible approach should ensure that participants are suitably remunerated according to the setting, context, and advice of their carers.

##### 2.3.2.1 The analysis

- Anonymised verbatim transcripts from participants will be analysed.
- Realist Logic Analysis: Identifying common themes, recommendations, and challenges across the qualitative data will inform further development of the CMOCs and programme theory from WS1. Additional CMOCs will be developed, where necessary to account for new relevant data. This analysis will highlight key areas for improvement and opportunities for trauma-responsive care.
- Stakeholder Interpretation Discussions: Collaboratively interpreting findings with stakeholders and discussing potential solutions. Their insights will help refine our theory and recommendations to ensure they address user needs.

Finally, we will draw upon the realist programme theory of the intersections between the effects and experiences of trauma, TRC, adversity, resilience, and residential care with the findings from the EBCD process to develop the new TRC intervention and implementation toolkit for WS3. We will hold events with the LEAG and SAB, as described in WS1, to gather feedback on the drafted new TRC intervention and implementation toolkit and make final amendments. Together, the perspectives offered through the interpretation of data presented to these groups will provide opportunities for meta-learning on the process, factors for decision making, and insights into how the intervention might operate within the children's home environment.

The residential nature of the homes and enthusiasm from the partners will ensure there are no delays with recruitment as all parties are already on-site. Additionally, if we need to extend this part of the research process, we can do, with little impact on WS3 as all activities are being undertaken in the same locations.

**WS2 Output:** A new TRC intervention with an implementation toolkit tailored for the unique context of children's homes, piloted and evaluated in WS3.

We envision WS1 and WS2 will inform:

- An attainment, improvement and monitoring framework, identified and developed through WS1 and 2, and will help set clear SMART goals for the home managers and staff.
- A theoretical induction in resilience, recovery, adversity, and TRC specifically in relation to children in care, taught to staff at all levels through three training days, team formulation sessions and ongoing supervision, supported by SP, EF, and the research team.
- The TRC intervention will be taught to staff, who will be supported in implementation through training, supervision, and support workshops. This process will also help the research team build the implementation toolkit around the intervention as it is used, collecting stories from practice, reflective case examples, and problem/solution-based learning examples.
- An integrity checklist, implemented through peer observation and support from the research team.
- An embedded schema for team formulation meetings and supervision for the frontline staff that will initially be run by members of the research team, learnt by the home staff, and then adopted so it can be continued after the pilot has finished.
- SP and EF are experienced trainers and consultants within children's homes, and will conduct the training, supervision, and team formulation sessions, supported by the research team and home managers.

#### 2.3.3 Realist evaluation

WS3a aims to further develop and test (confirm, refute or refine) the programme theory from WS1 and our new TRC intervention and implementation toolkit across the homes, evaluated using mixed-method Realist Evaluation (47, 48). Data collection will include both regular and in-depth qualitative data, routinely collected data about children and young people, and quantitative data (including validated data collection tools). Our hypothesis is that the intervention will flexibly support key mechanisms to bring about observable change in terms of creating a culture of resilience, community cohesion and community spirit, as well as enhancing individual wellbeing, attainment, and resilience in the face of stressors, transitions, and adversity.

##### 2.3.3.1 Data collection will include

- Personal demographics of all participants at the start of the study or when a new member of staff or young person joins each home.
- Quantitative data to track children's development and wellbeing at the start and end of the pilot, or when the child leaves the home if before the end of the pilot.
  - The strengths and difficulties questionnaire (SDQ) (49) a 25-item beginning and end.
  - Revised Children's Anxiety and Depression Scale (RCADS) a 47-item beginning and end.
  - The Bounce Forwards Scale, which has 5 domains that align with expected changes resulting from a TRC intervention- 34 item beginning and end.
  - The emotional support scale from the Paediatric Social Relationship Scales (50) −7+1 item beginning, middle and end.
  - The care environment scale from the CREATE Report Card (51) −5+2 item beginning and end.
  - The Professional Quality of Life Scale (ProQOL) (52) will be completed by all participating staff members, at the start and end of the pilot.
- Tabulated data will be gathered from the monthly routine monitoring data (RMD), which all children's homes collect in relation to child development, educational attainment, incidents of concern, and their wellbeing.
- A brief TRC self-assessment reflective exercise will be undertaken with members of staff at the start and end of the pilot. This will be created by the research team during WS1 & 2, to identify changes and explore relationships between the implementation of the TRC intervention and compassion satisfaction, burnout, and secondary traumatic stress. beginning and end.
- A bi-monthly pen portrait completed by key workers and home managers, documenting intervention integration, integrity, and implementation, will be developed in Reflective Practice Groups (RPGs).
- Realist qualitative interviews and/or reflective group discussions with young people and the workforce to confirm, refute or refine intervention theory will be conducted towards the end of the pilot intervention, or earlier where possible if a young person or member of staff is due to leave their placement or employment before the end of the pilot.
- Previous research undertaken by the authors across the care sector has demonstrated that the workforce prefers an anonymised and flexible feedback option to share experiences. Therefore, an anonymous Qualtrics form will be available throughout the pilot to provide an opportunity for ongoing feedback.

**Setting:** Four local authority homes and two privately run homes in the Northwest of England have elected to collaborate on this project to enhance their service delivery. The initial phases of the EBCD approach and baseline assessments taken at the start of the evaluation will inform interpretations of the findings to support theoretical transferability across a fractured and under-performing sector.

##### 2.3.3.2 Data analysis

Qualitative data analysis software (*N*Vivo) will be used to manage qualitative transcribed verbal and text data. We will analyse the data using the same realist logic of analysis as in WS1 and WS2. Our analysis will enable us to further confirm, refute or refine our understanding of the implementation and performance of the new intervention, which will be captured in a refined programme theory.

Quantitative data will be analysed using descriptive statistics, regression analysis and pre- and post-test methods, which will be the likely suitable analyses based on expected participant numbers and frequency of administration of measures. However, this approach will be reviewed at regular points during the research process. Where relevant, quantitative data will be drawn on to inform programme theory development and testing.

#### 2.3.4 Refinement of intervention and implementation toolkit

The increased understanding in our programme theory from WS3a will inform the refinement of the intervention and implementation toolkit, reflecting learning throughout the evaluation. The intervention and implementation toolkit revisions will be presented to staff and young people across the homes for feedback before being finalised. The final draft will then be presented to the LEAG and SAB for final edits.

1. Refinement Workshops with Frontline Workers (*N* = 20) and PPIE Groups
  - Purpose: To refine the intervention elements and gather practical insights into how TRC can be effectively implemented within children's homes.
  - Activities:
    - Facilitated sessions to discuss practical challenges, adaptability, and barriers in the proposed TRC protocol.
    - Evaluation of toolkit components, with feedback on usability, prioritisation and relevance to daily care practices.
2. Interpretation Focus Groups with Stakeholders
  - Purpose: To explore feasibility and ensure that the toolkit's design supports key mechanisms during practical implementation while remaining adaptable to specific contexts.
  - Activities:
    - Detailed discussions on toolkit integrity during application, including role-playing or scenario-based testing to evaluate flexibility.
    - Analysis of potential risks and adaptations required to maintain TRC principles while adjusting to specific organisational or situational demands.
3. Programme Theory Development
  - Purpose: To finalise the programme theory by incorporating insights from the workshops and focus groups, achieving a thorough understanding of the “what works, for whom, how, why, and when” of the TRC intervention.
  - Activities:
    - Consolidation of insights and case examples from frontline staff and PPIE participants on specific elements of TRC interventions that are effective in supporting children's resilience.
    - Examination of causal mechanisms (e.g., psychological safety, trust-building) within TRC practices and conditions under which these mechanisms best support outcomes.

**Outputs:**

1. A well-refined intervention and implementation toolkit that is based on tested generative causal theory and grounded in frontline experience and practical needs, ensuring its usability and acceptability in real-world settings.
2. A new approach for TRC in children's homes that is shaped by toolkit feasibility, ensuring that fidelity to trauma-responsive principles can be maintained despite variable contexts and situational challenges.
3. A comprehensive programme theory of change that articulates how the TRC intervention can be successfully implemented in children's homes, guiding stakeholders on factors impacting its effectiveness and sustainability in diverse contexts.

##### 2.3.4.1 Outcomes/endpoints

Primary Outcomes

- Wellbeing and resilience of children and young people
- Staff wellbeing and professional satisfaction
- Embedded TRC intervention

Secondary Outcomes

- Quality of therapeutic relationships
- Sense of community within homes
- Organisational change and service quality

#### 2.3.5 Data collection and measurement

##### 2.3.5.1 Calibration, validation, and reliability

Standardised administration protocols will be followed to ensure consistency across sites and time points. The Strengths and Difficulties Questionnaire (SDQ), Revised Children's Anxiety and Depression Scale (RCADS), and Professional Quality of Life Scale (ProQOL) have demonstrated strong internal consistency, test-retest reliability, and construct validity in youth and adult populations. The SDQ is widely used in UK youth mental health services, while the RCADS is validated across diverse clinical and community settings.

##### 2.3.5.2 Data collection schedule

Data will be collected at baseline (Month 1), mid-point (Month 6), and endpoint (Month 12) where possible, depending upon when children and young people start their placement (i.e., start of placement is baseline) or when staff start in post (i.e., start of post is baseline). Routine monitoring data (RMD) will be collected monthly. Narrative data will be gathered bi-monthly, and feedback events will be held at three key time points.

##### 2.3.5.3 Definitions and measurement instruments

###### 2.3.5.3.1 Assessment instruments and suitability

- SDQ: 25 items; 10–15 min. Suitable for ages 11–17. Validated for behavioural and emotional screening in youth.
- RCADS: 47 items; 10–15 min. Assesses anxiety and depression across six subscales. Validated for ages 8–18.
- Bounce Forwards Scale: 34 items; 10–15 min. Measures resilience and adaptive functioning.
- The Emotional Support Scale: 8 (7 + 1) items; 2–3 min. A subscale of the Paediatric Social Relationship Scales. Measures availability of emotional support. With an additional question about access to an advocate. Validated for ages 8–18.
- The Care Environment Scale (Adapted): 7 (5 + 2) items; 2–3 min. A subscale of the CREATE Report Card. Measures physical and emotional perceptions of the care environment. With two previously used and tested additional questions about global satisfaction with the environment and inclusion in decision-making.
- ProQOL: 30 items; 5–8 min. Assesses compassion satisfaction, burnout, and secondary traumatic stress in professionals.
- TRC Self-Assessment Tool: Developed in Workstream 1 to support reflective practice.
- TRC Implementation Domains Tool: Adapted from Scotland's national TRC framework; assesses fidelity across 10 domains.

###### 2.3.5.3.2 Narrative and qualitative data

Narrative data will be collected through semi-structured interviews, pen portraits, reflective group discussions, and anonymous written feedback via Qualtrics. These data will be analysed using realist logic to identify context-mechanism-outcome (CMO) configurations and inform programme theory development and testing, particularly regarding the identification of universal key principles that should be supported by interventions.

- Qualitative Interview Schedule (key staff): Adapted from Scotland's national TRC Implementation Domains Tool; assesses changes across 10 domains.
- Implementation Monitoring Template (staff): Developed in Workstreams 1&2 to gather brief qualitative data relating to effects and experiences of the intervention and implementation.
- Schedules for Realist Qualitative Interviews and/or Reflective Group Discussions (CYP): Developed in Workstreams 1&2 to confirm, refute or refine intervention theory.
- Pen Portrait Template: To collect information about TRC approaches for individual CYP.
- Anonymous Qualtrics Questionnaire: Developed in Workstreams 1&2 to collect anonymous written feedback.

###### 2.3.5.3.3 Statistical analysis plan

**Statistical Methods for Primary/Secondary Analyses**

Quantitative data will be analysed using descriptive statistics, pre- and post-test analysis and regression modelling.

**Subgroup/Sensitivity/Exploratory Analyses**

Subgroup analyses will explore differences by age, role, and setting. Exploratory analyses will inform refinement of programme theory.

**Software, Version, Libraries and Packages**

NVivo will be used for qualitative analysis, SPSS or equivalent for quantitative analysis.

**Significance Thresholds, Multiple Comparisons, Corrections**

Due to the exploratory nature of the study, formal significance thresholds are not specified. Findings will be interpreted within the realist evaluation framework.

###### 2.3.5.3.4 Monitoring, safety, and quality control

####### 2.3.5.3.4.1 Monitoring plan and audit

Monthly team meetings will review protocol adherence, data quality, and adverse events. A protocol deviation log will be maintained.

####### 2.3.5.3.4.2 Adverse events and harms reporting

Throughout the participants' involvement in the study, best practice, professional guidelines, and local NHS policies for monitoring mental state and risk for participants will be followed and will be facilitated by close liaison with clinical teams. Any adverse event (AE), clinically significant deterioration in the participants' mental state or change in risk information will be promptly communicated to responsible clinicians to ensure appropriate monitoring and provision of support.

Any AE observed over the course of the research will be documented and reported according to bespoke standard operating procedures (SOPs) that will fully comply with appropriate Health Research Authority (HRA) safety reporting procedures for non-CTIMP studies, Sponsor's requirements, and local Research and Development (R&D) policies of participating NHS organisations. For example, all research contacts will be recorded in clinical notes, and signed consent forms will also be uploaded/attached to clinical notes.

The occurrence of AEs will be monitored and systematically recorded by study staff. Research workers may become aware of an AE in a variety of ways, including participants prompted or unprompted disclosure, information received from responsible clinicians, information extracted through clinical notes and usual monitoring of the participants’ mental health and welfare as part of therapy sessions delivered as part of the trial. To ensure active surveillance of harms, at each follow-up assessment, the research workers will actively check for the occurrence of specific AEs using a structured checklist completed with the participant.

AEs are defined in line with standard HRA guidance as any untoward medical occurrence, unintended disease or injury, or untoward clinical signs in participants, whether or not related to the treatment, which require additional support or input from health professionals. Any clinically significant increase in presenting difficulties reported by participants (i.e., operationalised as an unresolved exacerbation in distress/mental health symptoms requiring increased involvement from the care team, e.g., a change in treatment plan) and reports of distress or complaints associated with therapy or other study procedure would also constitute AEs.

AE forms will be sent to the project CI (or another clinically qualified person with delegated responsibility) and assessed for:

- *Severity* (i.e., classified as mild, moderate, and severe according to the impact of the event on the person at the time, irrespective of whether the event also meet 'seriousness' criteria).
- *Relatedness* (i.e., whether the event resulted from administration of any of the research or therapy procedures, according to available information, e.g., temporal proximity to a study procedure; according to the report of the participant and the opinion of the clinical team).
- *Expectedness* (rated only in cases where the event is judged as related to the study procedures and intervention, and pertaining to whether the nature and severity of the observed reaction appear inconsistent with those expected from the study procedures.
- *Seriousness* (i.e., whether the outcome of the event meet criteria for a SAEs, including death and life-threatening events, incidents which acutely jeopardise the health or psychological well-being of the individual, events resulting in immediate hospital admission and/or persistent or significant disability or incapacity, and events resulting in injury requiring immediate medical attention, including A&E visits for mental health reasons).

Only Significant AEs (SAEs) judged to be unexpected and related to the study will be reported to the REC as per standard HRA procedures, within 15 days of the Chief Investigator (CI) first becoming aware of the event. This means the research ethics committee (REC) will be notified based on the initial report, even if the final report is pending. All reportable SAEs will be reported to the Sponsor in accordance with timelines and procedures mandated by Sponsor-specific guidelines and SOPs.

All completed AE forms will be stored locally in site master files, and a central AE log will be maintained as per HRA guidance to ensure effective safety monitoring. Throughout the trial, AEs and SAEs will be regularly audited at monthly team meetings to monitor trends in AE/SAE and their implications for the ongoing delivery of the study procedures. The Sponsor and Funder will immediately be notified on receipt of any information that raises material concerns about safety of the study procedures and interventions. To manage risks and safeguarding concerns, the research team ill follow the procedure outlined in Figure 2.

**Figure 2:**
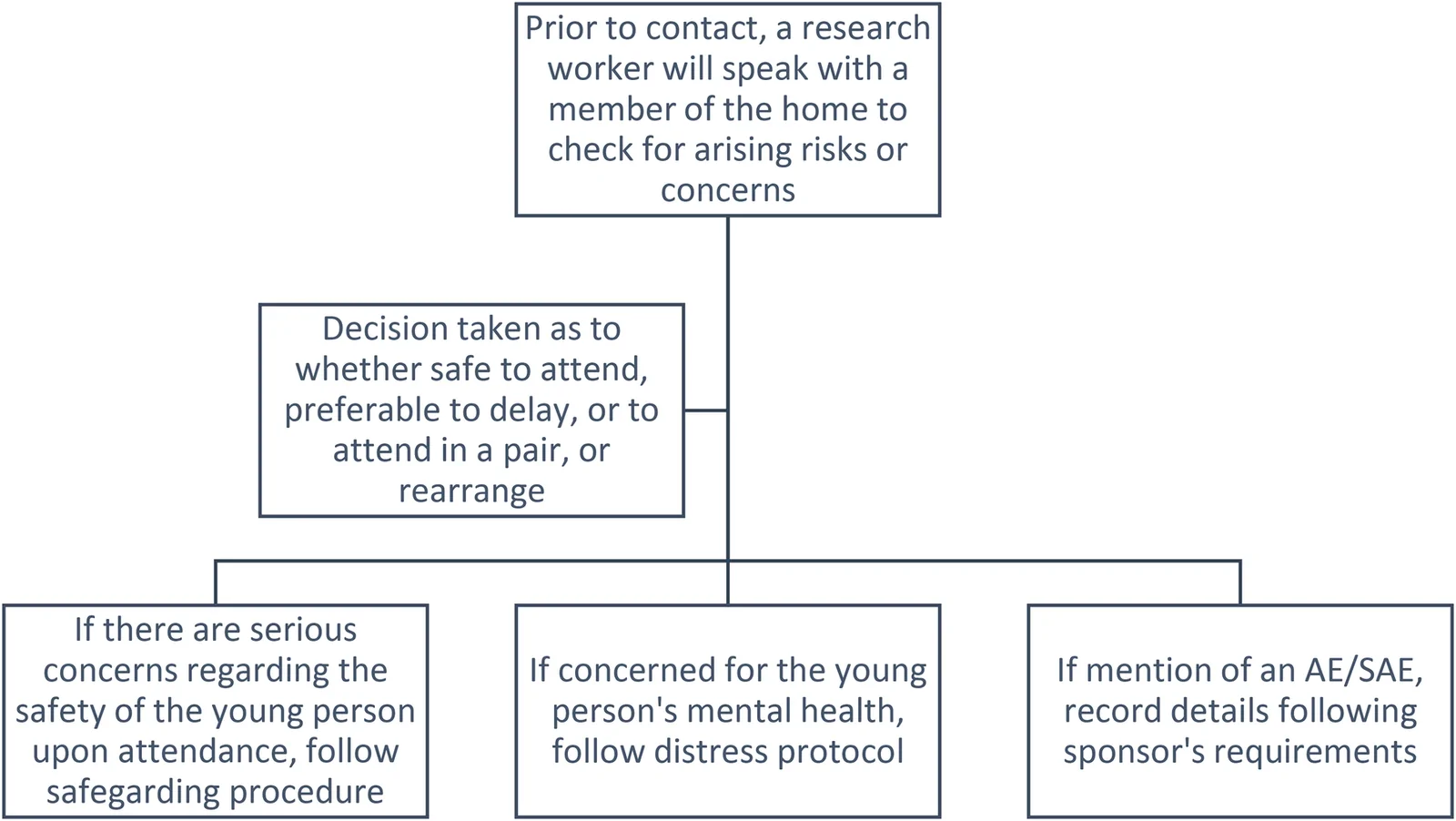
Risk management and notification process.

Any required urgent safety measures (i.e., steps taken by the CI and/or research team in the event that there is an immediate risk to a participant or participants, without the prior approval of the NHS REC/HRA) will be notified by the CI to the sponsor and REC immediately by telephone or email, as appropriate, and then followed-up with a modification within 3 days outlining the measures that have been taken and its rationale where changes to procedures have been required. A copy of the modification will be submitted to the Sponsor for expedite review and sponsor authorisation of the amendment before being submitted to the NHS REC/HRA.

####### 2.3.5.3.4.3 Participant confidentiality and data protection

The processing of all personal and research data will be in full compliance with the Data Protection Act 2018 and the European Union's General Data Protection Regulation (GDPR). Any personal information, except for participant's who have chosen an identifiable option for the filmmaking, will be deleted and/or safely destroyed at the end of the study e.g., through confidential waste management services available at our HEIs and NHS organisation. This will include pseudonymisation keys, i.e., data will be fully anonymised at the end of the study. All anonymised research data will be kept in anonymised format and retained for a minimum of five years following the end of the study. All final locked datasets will be kept in encrypted files on robust and automatically backed up on NHS Trust servers.

With regards to the films, a Data Protection Impact Assessment (DPIA) data transfer agreement will be drawn up between the Sponsor and collaborating organisations. In short, this agreement will outline that the film data is captured via MMU's cameras using password protected, encrypted SD cards. Ethical procedures to data handling outline that the filmed interview data is transferred on location straight after filming from the camera SD cards to MMU's editing suit. This is again using a password protected, unique identifier to be edited by the filmmaker EC. At this point, the film data is deleted from the SD cards. The un-edited films are then backed up and saved onto a designated RDS drive until the films are created. When created and approved by the participants and the research team, these unedited films are then deleted from the RDS Drive and only the approved edited versions are kept.

Participants over 16 years, will have the opportunity to co-edit their individual case study films with the research team before it gets integrated into the child's collective voice film. At this stage they will also be given a second chance to change their anonymity choice should they wish to and to edit out anything they do not want to be included in the final film. The EBCD films will be edited using FinalCut ProX software using the themes emerging from the thematic analysis. The individual case study films will be uploaded to University Tube for the purpose of research dissemination and will be active if the webpage is active. This will be password protected and locked, only accessible to be viewed and shared by the University co-applicants on the team. Storage of the collective voice films will be reviewed annually by the project CI (SP) for relevance as per GDPR guidance. If anyone who has chosen an identifiable option for filming wishes to withdraw after the EBCD final collective child's voice is made and uploaded, the research team will delete their individuals case study film (if aged over 16) remove from the University Tube site and fully anonymise that participant's data from the age specific and final collective child's voice film before uploading the amended version. Robust data security measures will be implemented throughout the study, in full compliance with national policies and relevant data management and information governance policies and procedures of the participating Higher Education Institutions and NHS organisations. Hard copies of participant questionnaire data and interview transcripts will be stored in safe lockable cabinets on Trust premises. Hard copies of signed consent forms will be stored in a similar way and will be kept separate from research data collected as part of the study. Signed consent forms will be stored in line with the requirements of the Sponsor. Study participant consent forms will be stored for five years after the study end date, and healthcare professional consent forms will be stored for 5 years after the study end date.

Any digital/electronic copies of research measures, interview transcripts and audio recordings will be encrypted and stored on secure and automatically backed up serves available at Sponsor sites. All research data will be pseudonymised, and unique study IDs will be used instead of participant names/Personal Identifiable Data (PID). Whenever possible, interviews will be conducted using recording devices enabling data encryption at the point of data collection, to provide additional data security. All interviews will be pseudonymised at the point of transcription, and all identifying details removed. Audio-recorded consent (including participants’ names) will be recorded on a separate audio file so that this information could not be directly linked with interview transcripts or audio-recordings. Digitally encrypted audio recordings of the interviews (but not identifying consent data, see above) will be transferred to an external company for transcription. To strengthen fidelity monitoring, a proportion of NEST-led sessions (e.g., reflective practice groups, supervision and formulation sessions) will be reviewed by trained members of the research or supervision team using the developing fidelity checklist, which will be one of the outputs for this co-production realist study. Fidelity assessments will be undertaken at regular intervals where possible and appropriate throughout the latter stages of the pilot and evaluation, to monitor adherence to the NEST Framework as it becomes more refined throughout the process. Where more than one rater is involved, inter-rater reliability will be assessed periodically to ensure consistency in fidelity ratings. Transcripts and fidelity materials will be stored by the research team using digitally encrypted files. Any audio or video recordings collected for supervision or fidelity purposes will be permanently deleted once reviewed and rated by the supervisors. All data will be fully anonymised at the end of the study through the destruction of pseudonymisation keys.

Transcripts will be returned to the central research team using digitally encrypted files. Any audio or video recording of therapy sessions undertaken for the purposes of supervision and treatment fidelity/adherence checks will not be retained and will be permanently deleted as once reviewed/rated by a therapy supervisor. Data will be fully anonymised at the end of the study by destroying pseudonymisation keys.

The transfer of research data amongst participating sites will be managed via a secure web-based database system hosted on Trust servers, or alternative safe data transfer systems approved by the Sponsor. Access to the database will be restricted to members of the project team involved in data entry and analysis, using an in-built secure system to grant access and data management privileges that can be authorised only by the project CI/Co-CI.

At the end of the study, all study data, the Project Master File, and all site files will be forwarded for archiving with the study Sponsor.

####### 2.3.5.3.4.4 Risks and benefits

Risks are minimal and mitigated through safeguarding protocols. Benefits include improved care quality and wellbeing for children and staff. All digital and face-to-face contact with research participants will be conducted in accordance with bespoke SOPs to manage any risk uncovered as part of the planned research assessments. These will comply with national and local policies for safeguarding children and vulnerable adults. In case our research assessments will uncover significant safeguarding issues or risks, participants' confidentiality may be breached to comply with safeguarding best practice and ensure the safety of all parties. This might involve disclosure of clinical and risk information to the participants' clinical teams and relevant safeguarding teams, as guided by local frameworks and policies for safeguarding children and vulnerable adults. All participants will be informed of the boundaries and limits of confidentiality at the onset of the evaluation.

The study will include the collection and discussion of sensitive topics, and some participants may find these upsetting or potentially distressing. In our experience, severe distress caused by the proposed research procedures will be highly unlikely. Nonetheless, to mitigate risk of distress, all contact with research participants will be conducted according to SOPs to manage assessments in a sensitive and respectful way. We will also follow tried-and-tested protocols for recognising and responding to potential signs of distress during and following contact with research participants. These procedures include, amongst other steps, 1) pausing of any data collection/interview procedures should a participant become distressed; 2) offering breaks and opportunities for reassurance; 3) reminding participants that their participation is voluntary and of their right to withdraw at any point, without any detriment to them; 4) procedures for signposting participants to appropriate sources of support or summon emergency services in cases of extreme risk to the participant or the public. All participants will be provided with debriefing information that will include the contact details of relevant local support services that participants could access in the event of a crisis. This debriefing document will be updated regularly to ensure that information and resources are as up to date as possible throughout the study.

All research workers contributing to data collection activities will receive regular supervision from a senior researcher within the team as well as access to line management supervision and other ad-hoc supervision and guidance from clinically qualified NHS professionals. All contacts with research participants will take place at pre-specified times agreed by project's CI or individual with delegated responsibility, and according to a “clinical cover rota” that will guarantee that RAs within the host research centre have prompt access to clinically qualified members of the research team for initial risk management advice.

It is expected that a considerable amount of contact with research participants will be via remote means (e.g., telephone or digital platforms/software approved by the participating NHS organisations, e.g., Microsoft Teams). Risks to the physical safety of the investigator are therefore minimal in these circumstances. Any necessary face-to-face contact will be conducted in full compliance with the lone working policies of the participating NHS Trusts and HEIs where the research workers and other research workers will be based, which will include locally adapted safety checking for lone workers SOPs.

A Co-investigator within the project team is an expert in Child Safeguarding and will advise the team on complex issues or ethical dilemmas that emerge during the study regarding safeguarding. In most instances, the local child safeguarding procedures will be followed, in collaboration with the regional Safeguarding Children Partnership.

### 2.4 Equality, diversity, and inclusion

The process of recruiting people to the young people's advisory group (YPAG), stakeholder advisory group, and research participants will be informed by an awareness of inclusive practices for research participation. We are committed to hearing from a diverse range of young people and stakeholders, which will help us assess the cultural sensitivity of our interpretations of data and implementation toolkit planning. We will draw on guidance and recommended practices within the Race Equality Framework, addressing the 50 questions attending to the five domains of the self-assessment. Further, benefitting from the anonymous feedback forum, we will employ the four components of the Patient-Public Partnership Model as a model to structure our presentations to and conversations with the LEAG and SAB to reflect upon our application of the Race Equality Framework within the context of the study. The team have engaged with training through the Proud Trust on promoting culturally sensitive engagement with young people identifying as LGBTQ+.

#### 2.4.1 Study status and timeline

The study started in December 2024, and participant recruitment is due to start in January 2026, with the pilot intervention running from April 2026 to April 2027. Data collection will take place across this same period. The overall study is expected to reach completion by July 2027.

The project will progress through several key milestones. Workstream 1, the realist review, ran from May to November 2025. Workstream 2, focusing on co-design and intervention development, is planned for January 2026 to April 2026. Workstream 3, which encompasses the pilot and its evaluation, will take place from April 2026 to April 2027. Following this, programme theory refinement, dissemination and impact activities will be carried out between May and July of 2027.

#### 2.4.2 Dissemination and data sharing

No professional writers will be involved in the production of the final project report and other peer-reviewed publications that will result from the research activities conducted as part of the project. Authorship of various project outputs will be informed by authorship criteria proposed by The International Committee of Medical Journal Editors or equivalent criteria endorsed by specific peer-reviewed journals where manuscripts will be submitted. Exact authorship decisions, including any time limits and review requirements by co-authors, will be agreed by the research team over the course of the project.

All publications and outputs arising from the project will comply with the NIHR's publication requirements, including advance output notifications to NIHR, standard NIHR funding statements and NIHR/disclaimers.

Following completion of the study, participants will be provided with an accessible summary of the study findings (if they consented to this). The findings of the project will be written-up as a series of papers to be submitted for publication in peer-reviewed journal. Further dissemination will be via conference presentations at national and international academic conferences, as well as training seminars, mainstream and social media, and accessible public forums (e.g., blogs and ACAMH) to share findings in a range of accessible mediums.

To disseminate and promote the intervention and toolkit across the sector, a multimedia dissemination package will include a short film, illustrated toolkit, implementation section with case studies, and stories from experience. We will work with the LEAG and SAB on integrated knowledge translation to ensure our materials are suitable for a range of audiences. In line with EBCD methodology, a celebration event will thank participants and promote our findings through all mainstream and social media channels to increase visibility and impact. A minimum of four high impact journal papers will be generated: (1) Rapid Realist Review, (2) EBCD commentary, (3) Realist Evaluation, (4) Intervention and Implementation Toolkit publication with policy and practice recommendations. We will present our findings to the Policy Team of the DfE and Ofsted to share key findings relevant across the sector. We will promote the toolkit though the Children's Homes Association to ensure uptake and further feedback across the sector. SP has provided information, upon invitation, to the Policy Team of the DfE regarding workforce training and support for residential care workers in recognition of current shortfalls. Accordingly, key stakeholders are already engaged in this process.

A large dissemination event will be held at a local venue, including webcast via Microsoft Teams/Zoom for an online audience, to disseminate findings from the study. All stakeholders will be invited and members of the LEAG and SAB will be invited to present their reflections. Young people from the homes and LEAG will be supported as is needed so they can participate, or not, as fully as they would like. Local and national press will be engaged with the event to promote the reach of the findings.

The NEST Study will generate a range of outputs designed to inform practice, policy, and future research. A policy document and practitioner briefing will be produced for frontline workers in children's homes, home managers, and directors, with an addendum tailored for commissioners. Findings from each workstream will be disseminated through high-quality peer-reviewed journal publications. To ensure accessibility, media outputs will be shared via open-access digital platforms such as Children & Young People Now, including a multimedia portfolio that features the implementation toolkit. Based on extensive Patient and Public Involvement and Engagement (PPIE), the toolkit will include the intervention protocol, stories from experience, case studies illustrating practical application, reflective practice insights, and visual summaries to support engagement with young people. Additionally, the toolkit will contain a section on research-based learning and “social stories” to guide future service development and research initiatives, including recommendations for overcoming common challenges. Sector-wide webinars will be hosted to share findings and promote uptake across residential care settings.

##### 2.4.1.1 Anticipated impact and future research

If the findings from the proposed study suggest that a future trial is warranted, we will design a feasibility Randomized Controlled Trial across local authority and privately owned homes in Northwest England to establish the parameters for a large-scale multi-site definitive trial. Currently, it is not clear how trial conditions could be operationalised within these environments, although this study will provide learning in relation to these methodological challenges.

#### 2.4.2 Preliminary research findings

Research to date highlights the critical need for tailored TRC in residential settings for cared-for children, encompassing the needs of children and young people, the workforce, and the wider organisational context. Children in care often present with complex trauma histories, requiring relationally attuned, emotionally safe environments that support co-regulation and recovery (53). The concept of *restorative parenting* has emerged as a key framework for delivering such care, emphasising relational repair and reflective practice (17). Staff wellbeing is also central to trauma-responsive care; high levels of work-related stress and emotional exhaustion can undermine therapeutic efforts, making systemic support and professional development essential (16, 54). The COVID-19 pandemic further exposed vulnerabilities in residential care, reinforcing the importance of trauma-responsive approaches to maintain stability and emotional safety during crises (16). Organisationally, embedding trauma-responsive principles requires a whole-system commitment to relational consistency, resilience-building, and reflective supervision (18). Moreover, preparing children and young people for transitions out of care must be trauma-responsive, recognising the long-term impact of early adversity and the need for continuity and support (19).

##### 2.4.2.1 Limitations of pilot data

Our existing pilot work is largely qualitative in design and takes place in England only, limiting generalisability across diverse care contexts. Sample sizes are often small, and children's voices are underrepresented, with a stronger focus on practitioner perspectives. Some findings are context-specific, such as those emerging during the COVID-19 pandemic, and may not reflect typical conditions. Additionally, there is a lack of longitudinal data to assess sustained outcomes, and limited empirical analysis of organisational structures and leadership influences on trauma-responsive implementation. These gaps point to the need for broader, more inclusive, and longitudinal research to inform tailored, system-wide approaches. Additionally, there is an urgent need to define TRC in the unique context of homes for cared for children, which this study addresses directly. Further, there is a need to include the voices of children in research and to employ systematic and robust approaches to the evaluation of the TRC pilot, which is achievable through realist methods.

## 3 Discussion

### 3.1 Possible challenges, success criteria, and mitigation strategies

A key success criterion for the NEST Study is the establishment of an effective interdisciplinary team. Considerable time was invested during the pre-application phase to build relationships, networks, and a collaborative research process with professionals and stakeholders across the children's residential care sector. This groundwork is expected to support sustained engagement and effective collaboration throughout the study.

Another factor is the development of a manageable range of initial programme theories in Workstream 1, which will be tested and refined through Workstreams 2 and 3. These theories will build upon existing frameworks, including the Nuffield Foundation's Principles of Care for Children with Complex Needs. The study design incorporates sufficient time and flexibility, supported by a skilled research team and dedicated Research Practitioner (RP), to ensure the timely and rigorous delivery of each workstream. The iterative nature of the study is intended to produce a robust, co-produced trauma-responsive care (TRC) intervention with the potential to improve outcomes for children, enhance staff wellbeing, and inform future sector development.

Recruitment is not anticipated to be a major challenge due to strong stakeholder interest and the recognised need for a trauma-responsive framework in children's homes. However, the team acknowledges the importance of engaging each young person and staff member individually and sensitively to discuss informed assent/consent. Should a critical mass of participants in any home withdraw the viability of that site's continued involvement will be reviewed with collaborating organisations. A contingency plan is in place, with a nearby local authority offering to host the study if needed.

Retention will be supported by the embedded nature of the research within the homes and a flexible, participant-centred approach. This includes offering interviews outside typical office hours and designing engaging, age-appropriate research activities. Staff engagement will be further incentivised through access to trauma-responsive training and supervision, which aligns with the strategic priorities of the local authorities and Integrated Care Systems.

To maximise impact, the study has engaged key stakeholders at all levels, including policy teams, commissioners, and care providers. Dissemination will be multimodal, including academic publications, policy briefings, webinars, and multimedia resources. A mailing list will be developed to share quarterly updates, and regular online consultation events will be hosted to maintain stakeholder engagement. These strategies aim to amplify the study's findings, support implementation, and contribute to systemic change in trauma-responsive residential care.

### 3.2 Potential impact of findings and future directions

The NEST Study has the potential to significantly advance trauma-responsive care in residential settings by providing a co-produced, contextually grounded intervention tailored for homes for cared for children. Findings will inform best practices for TRC with care-experienced young people and frontline staff, while enhancing wider staff wellbeing and organisational culture. The implementation protocol and refined programme theory will offer practical guidance for care providers, commissioners, and policymakers, contributing to sector-wide improvements in care quality and transparency. Dissemination through multimedia outputs, policy briefings, and academic publications will ensure accessibility and uptake across diverse audiences. Ultimately, the study aims to lay the groundwork for a future multi-site trial and broader systemic change in trauma-responsive residential care for children and young people.
